# Reduced expression of *BAX* is associated with poor prognosis in patients with epithelial ovarian cancer: a multifactorial analysis of *TP53*, *p21*, *BAX* and *BCL-2*

**DOI:** 10.1054/bjoc.2001.2101

**Published:** 2001-09-01

**Authors:** M Schuyer, M E L van der Burg, S C Henzen-Logmans, J H Fieret, J G M Klijn, M P Look, J A Foekens, G Stoter, E M J J Berns

**Affiliations:** Department of Medical Oncology (Division of Endocrine Oncology); Department of Pathology, University Hospital Rotterdam/DDHK, The Netherlands

**Keywords:** ovarian cancer, prognosis, *TP53*, *BAX*, *BCL-2*, *p21*

## Abstract

Traditional clinicopathological features do not predict which patients will develop chemotherapy resistance. The *TP53* gene is frequently altered in ovarian cancer but its prognostic implications are controversial. Little is known on the impact of TP53-downstream genes on prognosis. Using molecular and immunohistochemical analyses we examined *TP53* and its downstream genes *p21* *BAX* and *BCL-2* in ovarian tumour tissues and have evaluated the results in relation to clinico-pathological parameters, clinical outcome and response to platinum-based chemotherapy. Associations of tested factors and patient and tumour characteristics were studied by Spearman rank correlation and Pearsons χ^2^ test. The Cox proportional hazard model was used for univariate and multivariate analysis. The associations of tested factors with response was tested using logistic regression analysis. *TP53* mutation, p21 and BCL-2 expression were not associated with increased rates of progression and death. Expression of TP53 was associated with a shorter overall survival only (relative hazard rate [RHR] 2.01 *P* = 0.03). Interestingly, when combining *TP53* mutation and expression data, this resulted in an increased association with overall survival (*P* = 0.008). BAX expression was found to be associated with both progression-free (RHR 0.44 *P* = 0.05) and overall survival (RHR 0.42 *P* = 0.03). Those patients who simultaneously expressed BAX and BCL-2 had a longer progression-free and overall survival compared to patients whose tumours did not express BCL-2 (*P* = 0.05 and 0.015 respectively). No relations were observed between tested factors and response to platinum-based chemotherapy. We conclude that BAX expression may represent a prognostic indicator for patients with ovarian cancer and that the combined evaluation of *BAX* and *BCL-2* may provide additional prognostic significance.   http://www.bjcancer.com © 2001 Cancer Research Campaign


*British Journal of Cancer* (2001) **85**(9), 1359–1367
© 2001 Cancer Research Campaign
doi: 10.1054/ bjoc.2001.2101, available online at http://www.idealibrary.com on IDEAL
http://www.bjcancer.com

### Reduced expression of *BAX* is associated with poor prognosis in patients with epithelial ovarian cancer: a multifactorial analysis of *TP53*, *p21*, *BAX* and *BCL-2*

**M Schuyer[1], MEL van der Burg[1], SC Henzen-Logmans[2], JH Fieret[2], JGM Klijn[1], MP Look[1], JA Foekens[1], G Stoter[1] and EMJJ Berns[1]**

[1]Department of Medical Oncology (Division of Endocrine Oncology); and [2]Department of Pathology, University Hospital Rotterdam/DDHK, The Netherlands

**Summary** Traditional clinicopathological features do not predict which patients will develop chemotherapy resistance. The *TP53* gene is frequently altered in ovarian cancer but its prognostic implications are controversial. Little is known on the impact of TP53-downstream genes on prognosis. Using molecular and immunohistochemical analyses we examined *TP53* and its downstream genes *p21*, *BAX* and *BCL-2* in ovarian tumour tissues and have evaluated the results in relation to clinico-pathological parameters, clinical outcome and response to platinum-based chemotherapy. Associations of tested factors and patient and tumour characteristics were studied by Spearman rank correlation and Pearsons $\chi^2$ test. The Cox proportional hazard model was used for univariate and multivariate analysis. The associations of tested factors with response was tested using logistic regression analysis. *TP53* mutation, p21 and BCL-2 expression were not associated with increased rates of progression and death. Expression of TP53 was associated with a shorter overall survival only (relative hazard rate [RHR] 2.01, $P$ = 0.03). Interestingly, when combining *TP53* mutation and expression data, this resulted in an increased association with overall survival ($P$ = 0.008). BAX expression was found to be associated with both progression-free (RHR 0.44, $P$ = 0.05) and overall survival (RHR 0.42, $P$ = 0.03). Those patients who simultaneously expressed BAX and BCL-2 had a longer progression-free and overall survival compared to patients whose tumours did not express BCL-2 ($P$ = 0.05 and 0.015 respectively). No relations were observed between tested factors and response to platinum-based chemotherapy. We conclude that BAX expression may represent a prognostic indicator for patients with ovarian cancer and that the combined evaluation of *BAX* and *BCL-2* may provide additional prognostic significance. © 2001 Cancer Research Campaign http://www.bjcancer.com

**Keywords**: ovarian cancer; prognosis; *TP53*; *BAX*; *BCL-2*; *p21*

Epithelial ovarian cancer is the most lethal gynaecologic malignancy in Western countries. In about 70% of the patients the disease presents at an advanced stage meaning that widespread intraperitoneal metastasis has already occurred. Despite a high overall clinical response rate to modern treatment, including debulking surgery and platinum-based chemotherapy, the reported 5-year survival rates for women with advanced ovarian cancer are still less than 25%. Although the majority of patients initially respond to chemotherapy, two thirds will die due to progressive disease that has become refractory to chemotherapy (Ozols et al, 1992). The prognostic characterization of ovarian cancer patients is currently routinely based on clinico-pathological parameters. These features, however, have been proven insufficient to define prognostic subgroups and to accurately predict response to chemotherapy. Identification of new prognostic factors to select patients with good or bad outcome might help to improve treatment.

The cytotoxic effect of cisplatin and its analogues is mediated through the interaction with DNA and formation of a variety of DNA adducts, followed by the induction of programmed cell death (apoptosis) and/or other mechanisms of cell death (Eastman, 1987,1990). It has been suggested that defects in the apoptotic pathway can result in chemotherapy resistance (Dive and Hickman, 1991). Many genes that either positively or negatively influence apoptosis have been identified among which are the p53 tumour suppressor gene product and members of the BCL-2 gene family of apoptosis regulators. The BCL-2 protein has been related to the inhibition of apoptosis (Hockenbery et al, 1990) and to prolonged cell survival following DNA-damage (Miyashita and Reed, 1993), whereas the BAX protein accelerates apoptosis and antagonizes the anti-apoptotic function of BCL-2 (Oltvai et al, 1993). BAX has been shown to homodimerize as well as to heterodimerize with BCL-2, and the balance between BAX and BCL-2 is crucial for survival following an apoptotic stimulus (Oltvai et al, 1993). In addition, recent studies have demonstrated that BCL-2 and BAX regulate not only apoptosis but also the cell cycle. Interestingly, the tumour suppressor gene TP53, which is mutated frequently (up to 50%) in epithelial ovarian tumours (Shelling et al, 1995) is besides cell cycle arrest, senescence and DNA repair, also implicated in apoptosis.

There is clear experimental evidence that TP53 aberrations play a critical role in the development and progression of ovarian cancer. With respect to ovarian cancer the prognostic value of TP53 is still controversial (Ferrandina et al, 1999 and references therein). Tumour heterogeneity, small numbers of tumours and

*Received 23 March 2001*
*Revised 16 July 2001*
*Accepted 7 August 2001*

*Correspondence to:* EMJJ Berns, Josephine Nefkens Institute (room Be 424), Department of Medical Oncology, PO Box 1738, 3000 DR Rotterdam, The Netherlands different techniques used for studying TP53 may be responsible for the reported inconsistencies about the prognostic value of TP53. Furthermore, miscellaneous chemotherapeutic regimens and different definitions of response make it difficult to evaluate the predictive value of response to chemotherapy. Since there still is a controversy with respect to the value of immunohistochemical or molecular based techniques (Casey et al, 1996; Schuyer et al, 1998; Skilling et al, 1996), we have chosen to utilize both approaches to study the clinical relevance of TP53. It is also not known how and to what extent *TP53* mutation affects the function of the protein. More insight could come from the study of its 'downstream genes'. To date, genes considered to be target genes of TP53 include BCL-2, BAX, topoisomerase II, multidrug resistance gene 1, insulin like growth factor binding protein-3, vascular endothelial growth factor and the cell cycle inhibitor $p21^{/WAF1/CIP1}$.

For the present study, we used molecular analyses and immunohistochemistry (IHC) to assess the TP53 status and IHC for p21, BAX and BCL-2 expression in epithelial ovarian tumours. Our aim was to analyse whether and which protein levels and/or mutational status are significantly related to patient or tumour characteristics, disease outcome and response to platinum-based chemotherapy. None of the factors investigated showed a clear association with response to platinum-based chemotherapy. However, we found that high BAX expression is a favourable prognosticator in univariate analysis. Our results suggest that BAX expression may be a prognostic indicator for patients with ovarian cancer.

**Table 1** Patient and tumour characteristics

| Patient and tumour characteristics | No. of patients |
|---|---|
| All | 102 |
| FIGO stage | |
| early (I-IIA) | 34 |
| advanced (IIB-IV) | 68 |
| Histologic type | |
| serous | 51 |
| mucinous | 13 |
| endometrioid | 17 |
| clear cell | 6 |
| mixed | 7 |
| poorly differentiated | 8 |
| Tumour grade | |
| 1 | 16 |
| 2 | 36 |
| 3 | 42 |
| unknown | 8 |
| Residual disease | |
| none | 42 |
| ≤ 1 cm | 28 |
| > 1 cm | 32 |
| Ascites | |
| present | 55 |
| absent | 46 |
| unknown | 1 |
| Response to chemotherapy* | |
| complete | 21 |
| partial | 4 |
| stable disease | 1 |
| progressive disease | 11 |
| not assessable | 40 |
| unknown | 4 |

* For 81 patients who received postoperative chemotherapy.

### MATERIALS AND METHODS

### Patients and samples

In the present study 102 ovarian tumour tissue specimens were included from patients of whom clinical follow-up data were available. The tumour tissues were obtained from patients who underwent primary surgery for ovarian cancer between 1988 and 1993 in hospitals in the southwestern part of the Netherlands. The median age of the patients at the time of surgery was 56 years (range 27–86). The disease was staged according to the International Federation of Gynaecology and Obstetrics (FIGO) (Kottmeier, 1976). Tissue biopsies were placed on ice immediately following surgery and stored in liquid nitrogen. Ninety-one samples were resected from the tumour within the ovary whereas 11 samples were obtained from the tumour extension to the omentum. Histological typing and grading were assessed on paraffin-embedded tissue specimens according to the classification of the World Health Organization (WHO). All tissue samples were reviewed by the same pathologist (S H-L). A detailed description of patient and tumour characteristics is listed in Table 1.

Three patients received radiation and 81 patients were treated with postoperative chemotherapy (Table 1). Platinum-containing therapy was given to 75 patients (71× Cyclophosphamide /cisplatin; 3× Cyclophosphamide/carboplatin and 1× taxol/ cisplatin). The remaining six patients received cyclophosphamide (2×) or melphalan (3×) and in one patient treatment was not specified. Clinical response was assessed according to the standard WHO response criteria (WH Organization, 1979). Twenty-one patients had a CR and in six out of these 21 patients complete remission was confirmed by second-look laparotomy: in three patients a pathologic CR and in the other three patients microscopic residual disease was observed. Response was not assessable in 40 patients, of which 27 had no macroscopic residual tumour after surgery and 13 had residual tumour less than 1 cm. The median follow-up for patients still alive was 78 months (range 2–120 months).

### Immunohistochemistry

Immunohistochemical staining was performed by a peroxidase labelled streptavidin-biotin-complex technique on 90 tumour samples. Five micron thick cryostat sections were fixed in 4% buffered formalin in case of TP53 and p21 or in acetone in case of BAX and BCL-2 and preincubated with 5% BSA-PBS for 5 min. Endogenous biotin was blocked with avidin and biotin (Avidin-biotin blocking kit, Vector Laboratories, Burlingame, CA, USA) for 10 min respectively. Sections were subsequently incubated with the appropriate mouse monoclonal for 1 h, i.e. for TP53 clone DO-1 (1:200, Santa Cruz Biotechnology, Santa Cruz, CA, USA) and DO-7 (1:100, Dako, Glostrup, Denmark); for p21 clone 2G12 (1:100, Pharmingen, San Diego, USA); for BAX clone 4F11 (1 mg/ml, 1:100; Beckman Coulter BV, Mijdrecht, The Netherlands); for BCL-2 clone 124 (1:100, Dako, Glostrup, Denmark). After washing with PBS (2× 5 min), biotinylated rabbit anti mouse Ig (diluted 1:200, Dako) in PBS containing 2% normal human serum, was applied for 30 min followed by an incubation with peroxidase labelled streptavidin-biotin-complex (Vecta Stain Elite Peroxidase kit, Vector Labs, Burlingame, CA, USA.) for 30 min. Visualisation of the antibodies was performed

 *© 2001 Cancer Research Campaign*

by incubating the sections with diaminobenzidine tetrahydrochloride (Fluka Chemie, Buchs, Switzerland) in the presence of 3% hydrogen peroxide for 10 min. All reactions were performed at room temperature. Sections were finally counterstained with Harris haematoxylin, dehydrated and mounted with Pertex. Sections were evaluated by two observers (S H-L and JHF). When possible, dependent on the amount of tumour tissue, 300 epithelial tumour cells were counted and results were given as the percentage positive tumour cells. Positive and negative controls were included. Expression data were divided into two categories: low (≤ 10% tumour cells) and high (> 10% tumour cells) TP53 expression (Casey et al, 1996; 79 samples evaluable); no p21 (no staining in any of the tumour cells) and p21 expression (> 0% tumour cells; 69 samples evaluable); low (≤ 75% tumour cells) and high (> 75% tumour cells) *BAX* expression (52 samples evaluable), as determined by isotonic regression analysis (Barlow et al, 1993); low (≤ 40% tumour cells) and high (> 40% tumour cells) BCL-2 expression (Bonetti et al, 1998; 88 samples evaluable). Samples were considered not evaluable if: (a) there was insufficient tissue; (b) sections did not contain 300 epithelial tumour cells or; (c) sections did not stain appropriately.

### DNA-isolation, PCR-SSCP and sequencing

The tumour tissue was pulverized in the frozen state to a fine powder, homogenized in phosphate buffer according to the EORTC procedure (EORTC group, 1980) and high molecular weight chromosomal DNA was isolated from 82 tumour specimens, of which 70 were also used in our immunohistochemical studies. Exons 5–8 of the *TP53* gene were analysed by single-strand conformation polymorphism (SSCP) and products with an altered electrophoretic mobility were analysed again and sequenced as described previously (Berns et al, 1996).

### Statistics

The strength of the associations between TP53, p21, BAX and BCL-2 used as continuous variables was tested by Spearman rank correlation or by Pearson's $\chi^2$-test for categorical variables. To test whether staining percentages for TP53 differed in tumour specimens with and without a mutation the Mann-Whitney test was used. A cut-off point for BAX expression was determined using isotonic regression analysis (Barlow et al, 1993). The relationship between patient and tumour characteristics and categorised TP53, p21, BAX and BCL-2 was tested using Pearson's $\chi^2$-test. In all analyses unknown ascites and grade were included as a dummy variable. Overall and progression-free survival probabilities were calculated by the actuarial method of Kaplan and Meier (1958) and the log-rank test was used to test for differences between groups and Cox proportional hazard model was for univariate and multivariate survival analysis. To evaluate whether BAX or TP53 contribute to the prognostic value of the classical prognostic factors in multivariate analyses for progression-free and overall survival, patients were stratified by age, FIGO stage, residual tumour rest and the presence of ascites. The likelihood ratio test was used to test between models with variables in-and excluded. The associations of variables with response to chemotherapy was tested using logistic regression analysis. These analyses were done with STATA statistical software (release 6.0 College Station, TX: Stata Corp). Two-sided *P*-values < 0.05 were judged statistically significant.

### RESULTS

### Immunostaining

The expression of TP53, p21, BAX and BCL-2 was studied by immunohistochemistry in primary ovarian tumours. Using the criteria described in the Methods section, 79 tumours were evaluable for TP53 immunostaining with DO1 antibody. In 31 specimens (39%) no nuclear immunoreactivity was detected. For the other 48 samples, 13 tumour specimens (16%) showed TP53 staining in less than 10% of tumour cells and staining in over 10% of tumour cells was observed in 35 (44%) tumours. These latter specimens were considered positive. Nuclear p21 expression was evaluated in 69 ovarian tumours. Nineteen specimens (28%) demonstrated p21 immunoreactivity and were defined as positive. BAX immunostaining was evaluable in 52 tumours. Cytoplasmic BAX staining was observed in 45 tumours and according to the cut-off point of 75% tumour cells 40 tumours (77%) were positive for BAX. Cytoplasmic BCL-2 immunostaining was evaluable in 88 tumours. Using the cut-off point of 40% positive tumour cells, 29 (33%) tumours were considered positive. No significant relationships were observed between TP53, p21, BAX or BCL-2 expression.

### TP53 gene alterations and relationship with immunostaining

Eighty-two epithelial ovarian tumour specimens were studied for *TP53* mutations by PCR-SSCP analysis (exons 5 through 8) and sequencing. Among 36 (44%) tumour samples with altered migration patterns, 32 gene mutations were detected. These included 22 missense point mutations resulting in an amino acid substitution, 7 nonsense mutations (in 6 tumours) and 2 frameshift deletions, leading to premature termination of the protein and a splice junction mutation leading to an altered transcription product. A significant correlation was observed between *TP53* mutation and immunohistochemical status ($P < 0.001$). As expected especially missense mutations correlated with positive immunostaining. Of the 20 tumour specimens with missense mutations that were both analysed by sequencing and immunohistochemistry, 19 specimens were found to be immunopositive (>10% positive tumour cells). Of the seven tumour specimens with non-missense mutations that were also analysed by immunohistochemistry, only the sample with a splice site mutation showed nuclear accumulation of the TP53 protein. Furthermore, 9 out of 36 mutation-negative specimens, which were analysed for TP53 expression, demonstrated immunopositivity although the level of expression was lower (median: 37%; range 15–90%) than in immunopositive tumour specimens with a confirmed *TP53* mutation (median: 90%, range 47–100%; $P = 0.0001$).

### Relationships with patient and tumour characteristics

A significant correlation was observed between *TP53* mutation or overexpression and advanced FIGO stage ($P = 0.008$ and 0.02 respectively) and between TP53 expression and the size of residual tumour after surgery, i.e. in tumours with a residual tumour rest larger than 1 cm TP53 expression was found more frequently ($P = 0.004$; Table 2). In addition, TP53 mutation and TP53 expression occurred more frequently in serous tumours. Moreover, *TP53* mutation and TP53 and BCL-2 expression were less frequently

**Table 2** Relationships of *TP53* mutation and TP53, p21, BAX and BCL-2 expression with patient and tumour characteristics

| | Gene mutation *TP53* | | | Protein expression | | | | | | | | | | | |
|---|---|---|---|---|---|---|---|---|---|---|---|---|---|---|---|
| | | | | TP53 | | | p21 | | | BAX | | | BCL-2 | | |
| **Factor** | ***n*** | **Positive (%)** | ***P*-value** | ***n*** | **Positive (%)** | ***P*-value** | ***n*** | **Positive (%)** | ***P*-value** | ***n*** | **Positive (%)** | ***P*-value** | ***n*** | **Positive (%)** | ***P*-value** |
| All | 80 | 31 (39) | | 79 | 35 (44) | | 69 | 19 (28) | | 52 | 42 (81) | | 88 | 29 (33) | |
| Age | | | | | | | | | | | | | | | |
| ≤ median | 44 | 16 (36) | | 42 | 17 (40) | | 37 | 8 (22) | | 25 | 18 (72) | | 46 | 16 (35) | |
| > median | 36 | 15 (42) | 0.63 | 37 | 18 (49) | 0.47 | 32 | 11 (34) | 0.24 | 27 | 22 (81) | 0.42 | 42 | 13 (31) | 0.70 |
| FIGO-stage | | | | | | | | | | | | | | | |
| early | 27 | 5 (19) | | 27 | 7 (26) | | 22 | 4 (18) | | 16 | 13 (81) | | 29 | 11 (38) | |
| advanced | 53 | 26 (49) | 0.008 | 52 | 28 (54) | 0.02 | 47 | 15 (32) | 0.23 | 36 | 27 (75) | 0.62 | 59 | 18 (31) | 0.49 |
| Tumour rest | | | | | | | | | | | | | | | |
| ≤ 1 cm | 53 | 17 (32) | | 54 | 18 (33) | | 46 | 9 (20) | | 34 | 28 (82) | | 59 | 20 (34) | |
| > 1 cm | 27 | 14 (52) | 0.09 | 25 | 17 (68) | 0.004 | 23 | 10 (43) | 0.04 | 18 | 12 (67) | 0.20 | 29 | 9 (31) | 0.79 |
| Ascites | | | | | | | | | | | | | | | |
| absent | 37 | 13 (35) | | 36 | 13 (36) | | 31 | 7 (23) | | 24 | 19 (79) | | 37 | 10 (27) | |
| present | 42 | 17 (40) | 0.63 | 42 | 22 (52) | 0.15 | 37 | 11 (30) | 0.51 | 28 | 21 (75) | 0.72 | 50 | 19 (38) | 0.28 |
| Histology | | | | | | | | | | | | | | | |
| serous | 38 | 18 (47) | | 39 | 21 (54) | | 35 | 10 (29) | | 27 | 19 (70) | | 45 | 18 (40) | |
| non-serous | 42 | 13 (31) | 0.13 | 40 | 14 (35) | 0.09 | 34 | 9 (26) | 0.85 | 25 | 21 (84) | 0.24 | 43 | 11 (26) | 0.15 |
| Grade | | | | | | | | | | | | | | | |
| 1 | 11 | 2 (18) | | 10 | 3 (30) | | 7 | 2 (29) | | 9 | 6 (67) | | 12 | 2 (17) | |
| 2 | 30 | 12 (40) | | 28 | 8 (29) | | 26 | 7 (27) | | 14 | 13 (93) | | 31 | 13 (42) | |
| 3 | 33 | 15 (45) | 0.27 | 35 | 19 (54) | 0.09 | 32 | 8 (25) | 0.98 | 27 | 19 (70) | 0.21 | 38 | 12 (32) | 0.28 |

© 2001 Cancer Research Campaign observed in more differentiated tumours, although these differences were not significant. Staining for p21 was more often observed in tumours of patients with residual tumour lesions larger than 1 cm ($P$ = 0.04). No other significant relations were found between patient and tumour characteristics and p21, BAX or BCL-2. This evaluation of tumours with multiple molecular markers sets up a situation in which multiple univariate analyses are performed. Table 2 looks at 30 different comparisons. When correcting for multiple comparisons (Bonferroni) a *P-value* of less than 0.01 can be considered significant, which implies that only *TP53* mutation with advanced disease and TP53 expression with suboptimally debulked disease retains significance.

### Progression-free and overall survival

In Cox univariate regression analysis, older age, advanced FIGO-stage, larger tumour rest and ascites at presentation were significantly associated with a short progression-free and overall survival (Table 3). Patients with grade 3 tumours had an early progression compared to patients with grade 1 tumours. No association was observed between p21 or BCL-2 expression and progression-free or overall survival in univariate analysis. Although patients with BCL-2 positive tumours appeared to have a longer progression free survival (median: 36 months) and overall survival (median: 59 months) compared to patients with BCL-2 negative tumours (median PFS: 27 and OS: 34 months), the differences were not statistically significant (Kaplan-Meier curves not shown). TP53 protein expression was found to be associated with a poor overall survival (Table 3). Patients with TP53 immunopositive tumours experienced an earlier death ($P$ = 0.03) compared to patients with TP53 negative tumours. Although there was a trend toward a poor progression-free survival in patients with TP53 immunopositive tumours, this difference was not statistically significant. Patients whose tumours showed *TP53* mutations also tended to have a worse progression-free and overall survival compared to patients whose tumours exhibited no mutations. However, these differences were not statistically significant. Patients with both mutation- and immuno-negative tumours had a better progression-free ($P$ = 0.07) and overall survival ($P$ = 0.008) compared to patients with either one or both parameters positive (not shown).

High BAX expression (> 75%) was associated with a favourable progression-free ($P$ = 0.05) and overall survival ($P$ = 0.03; Table 3 and Figures 1A and B). We also analysed the BAX data using a cut-off value of 40% and the Kaplan Meier curve showed a similar trend ($P$ = 0.05 for overall survival). The median progression-free and overall survival of patients in the high BAX group was 27 and 54 months respectively compared to 11 and 18 months in the low BAX group. In an exploratory subgroup analysis in patients with *advanced* disease ($n$ = 32 for PFS; $n$ = 35 for OS), BAX expression was also significantly associated with a longer progression-free (RHR: 0.40; 95% CI: 0.17–0.95; $P$ = 0.04) and overall survival (RHR: 0.43; 95% CI: 0.19–0.97; $P$ = 0.04). Since BAX and BCL-2 are antagonists and known to form heterodimers, we studied whether the combined evaluation of BAX and BCL-2 expression provided additional information on overall or progression-free survival. All patients with a low expression of BAX in their tumours had a similar poor survival, irrespective of BCL-2 status. Unexpectedly, of the patients with BAX positive tumours, those simultaneously expressing BCL-2 showed a longer progression-free and overall survival compared to patients whose tumours did not express BCL-2 (Figures 1C and D).

### Multivariate analysis for progression-free and overall survival

A possible independent prognostic significance of BAX and TP53 expression was examined by Cox multivariate analysis for progression-free and overall survival. Univariate analysis (Table 3) showed that age, FIGO stage, residual tumour rest after cytoreductive surgery, and the presence of ascites are factors that strongly predict disease outcome and survival. This has also been described by van der Burg et al (1996) and Neijt et al (1991). In multivariate analyses patients were therefore stratified by these factors. For the multivariate analysis for progression-free survival BAX and grade, both significantly related with progression-free survival in univariate analysis, entered the model. Only BAX expression tended to predict progression (RHR = 0.35; CI = 0.11–1.11; $P$ = 0.075). For the multivariate analysis for overall survival BAX or TP53 expression entered the model but neither were found to be independently associated with survival.

### Response to platinum-based chemotherapy in advanced disease

Of the 68 patients with advanced stage (IIB-IV) disease, 58 patients (FIGO stage IIB/IIC: 3 patients and stage III-IV: 55 patients) received platinum containing first-line chemotherapy. For three patients response was unknown (stage IIC, 1 patient), 21 patients showed no progression, 19 patients achieved a complete response, four a partial response, one patient had stable disease and 10 patients experienced progressive disease, thus the overall percentage that experienced progressive disease was 19% (10 out of 55 patients). In an exploratory analysis we studied the association of the markers investigated with response to platinum-containing combination therapy in advanced stage disease. There was no significant association between response to chemotherapy (complete or partial response versus stable or progressive disease) and p21, BAX, BCL-2 and TP53 expression or *TP53* mutation.

### DISCUSSION

Besides the classical prognostic factors, it would be beneficial for patients with ovarian cancer if there were additional tumour-associated markers that could more reliably predict the rate of progression and/or the efficacy of response to chemotherapy. In the present study we determined the relationship between *TP53* mutation and expression and the expression of its downstream genes (i.e. the cell cycle inhibitor p21, the cell death agonist BAX and its antagonist BCL-2) and evaluated the outcome in relation to patient and tumour characteristics, survival and response to platinum-containing chemotherapy in patients with ovarian cancer.

In the present study only TP53 expression was found to be significantly ($P$ = 0.03) associated with a poor overall survival, in univariate analysis. However, as shown in multivariate analysis, TP53 expression was not independently associated with survival. Interestingly, patients whose tumours were both mutation-and immunonegative had a clear survival advantage compared to patients whose tumours had either a mutation and/or TP53 overexpression (RHR: 2.85, $P$ = 0.006). The observation that the combination of both TP53 expression and mutation data results in a stronger prediction of outcome has recently been reported by Wen et al (1999) as well. However, more studies are needed to verify the prognostic value of the combined expression and

*© 2001 Cancer Research Campaign*

**Table 3** Cox univariate analysis of progression-free and overall survival

| Factor* | Progression-free survival | | Overall survival | |
|---|---|---|---|---|
| | *P*-value | RHR (95% CI)† | *P*-value | RHR (95% CI)† |
| Age (continuous) | 0.03 | 1.02 (1.00–1.04) | 0.004 | 1.03 (1.01–1.05) |
| > 56 vs ≤ 56 years | 0.02 | 1.94 (1.12–3.35) | 0.001 | 2.68 (1.53–4.69) |
| FIGO-stage | | | | |
| advanced vs early | < 0.001 | 8.80 (3.72–20.82) | < 0.001 | 24.95 (6.05–102.88) |
| Tumour rest | | | | |
| >1 cm vs ≤ 1 cm | < 0.001 | 5.53 (3.08–9.94) | < 0.001 | 6.01 (3.40–10.64) |
| FIGO-stage / Tumour rest | | | | |
| advanced/ ≤ 1 cm vs early | < 0.001 | 6.33 (2.57–15.61) | < 0.001 | 17.77 (4.19–75.37) |
| advanced/ > 1 cm vs early | | 18.17 (7.10–46.48) | | 42.71 (10.02–182.06) |
| Ascites | | | | |
| present vs absent | 0.01 | 2.11 (1.20–3.72) | < 0.001 | 3.05 (1.68–5.56) |
| Histology | | | | |
| non-serous vs serous | 0.21 | 0.71 (0.41–1.21) | 0.24 | 0.73 (0.43–1.24) |
| Grade | | | | |
| grade 2 vs grade 1 | 0.03 | 1.95 (0.66–5.80) | 0.18 | 1.27 (0.50–3.21) |
| grade 3 vs grade 1 | | 3.27 (1.14–9.36) | | 1.96 (0.81–4.75) |
| *TP53* mutation | | | | |
| mutation vs no mutation | 0.10 | 1.69 (0.91–3.13) | 0.10 | 1.66 (0.91–3.05) |
| TP53 expression | | | | |
| > 10% vs ≤ 10% | 0.18 | 1.53 (0.82–2.83) | 0.03 | 2.01 (1.08–3.74) |
| p21 expression | | | | |
| > 0% vs 0% | 0.32 | 1.40 (0.73–2.68) | 0.49 | 1.26 (0.65–2.47) |
| BCL-2 expression | | | | |
| > 40% vs ≤ 40% | 0.37 | 0.75 (0.39–1.42) | 0.31 | 0.72 (0.38–1.37) |
| BAX expression | | | | |
| > 75% vs ≤ 75% | 0.05 | 0.44 (0.19–1.01) | 0.03 | 0.42 (0.19–0.93) |

PFS (OS): $n$ = 94 ($n$ = 101) for clinico-pathological variables except for ascites and grade (1 respectively 8 missing values); $n$ = 48 ($n$ = 51) for Bax expression; $n$ = 82 ($n$ = 87) for Bcl-2 expression; $n$ = 65 ($n$ = 68) for p21 expression; $n$ = 74 ($n$ = 78) for TP53 expression; $n$ = 73 ($n$ = 79) for *TP53* mutation. † RHR: relative hazard rate with 95% confidence interval (CI).

mutation data. With respect to treatment, in this relatively small group of advanced ovarian cancer patients no association between TP53 expression or mutation and response to platinum-based chemotherapy was found. This is in agreement with data from previous immunohistochemical-based studies (Hartmann et al, 1994; Herod et al, 1996; Rohlke et al, 1997; van der Zee et al, 1995) but in contrast to other studies using either immunological (Dong et al, 1997; Ferrandina et al, 1999) or molecular-based techniques (Buttitta et al, 1997; Righetti et al, 1996).

The presence of a TP53 aberration is not informative for the biological function of TP53, thus additional information could be provided by the study of downstream genes and although TP53 regulates the expression of these genes in vitro (el-Deiry et al, 1993, 1994; Miyashita et al, 1994; Miyashita and Reed, 1995), we could not confirm any correlation between *TP53* mutation or expression and expression of p21, BCL-2 or BAX in ovarian tumour specimens. This is consistent with findings from several other studies (Baekelandt et al, 1999a, 1999b; Diebold et al, 1996; Herod et al, 1996; Marone et al, 1998) although an inverse correlation between TP53 and BCL-2 (Henriksen et al, 1995) and between TP53 and p21 (Anttila et al, 1999) has also been reported in ovarian tumour tissues. The lack of a correlation between TP53 and its downstream genes may reflect the fact that expression of these genes is also regulated by TP53-independent pathways.

Expression of p21 was only associated with tumour rest in this study. This is in agreement with a recent report that also failed to find an association between p21 expression and prognosis or response to platinum-based chemotherapy in specimens from stage III ovarian cancer patients (Baekelandt et al, 1999a). In contrast to these findings Anttila et al (1999) reported that low p21 expression, using a polyclonal antibody, is a marker of poor overall survival.

No statistically significant association between BCL-2 expression and survival was found. However, patients with increased BCL-2 expression tended to have a better progression-free and overall survival compared to patients with low BCL-2 expression in their tumours. Several studies have correlated BCL-2 with a survival advantage in ovarian cancer (Baekelandt et al, 1999b; Diebold et al, 1996; Henriksen et al, 1995; Herod et al, 1996) but failed to find an association with overall response to chemotherapy (Baekelandt et al, 1999b; Herod et al, 1996). In contrast, BCL-2 expression has also been reported to be associated with a poor prognosis and resistance to chemotherapy (Kassim et al, 1999; Mano et al, 1999). Since BCL-2 is thought to function as an anti-apoptotic protein, a correlation between BCL-2 expression and a favourable outcome may seem paradoxical. This inhibition of tumour cell growth by BCL-2 has also been observed in certain solid tumour cell lines (Pietenpol et al, 1994) and in breast cancer as well (Gasparini et al, 1995). Furthermore, it has been suggested that BCL-2 plays a role in the suppression of angiogenesis (Koukourakis et al, 1997). Thus BCL-2 may have different functions in normal differentiated and in cancer cells. Moreover, there is also evidence that BCL-2 functions as a proapoptotic protein in some circumstances since over-expression of the BCL-2 protein has been shown to increase the half-life of the BAX protein (Miyashita et al, 1995).

*© 2001 Cancer Research Campaign*

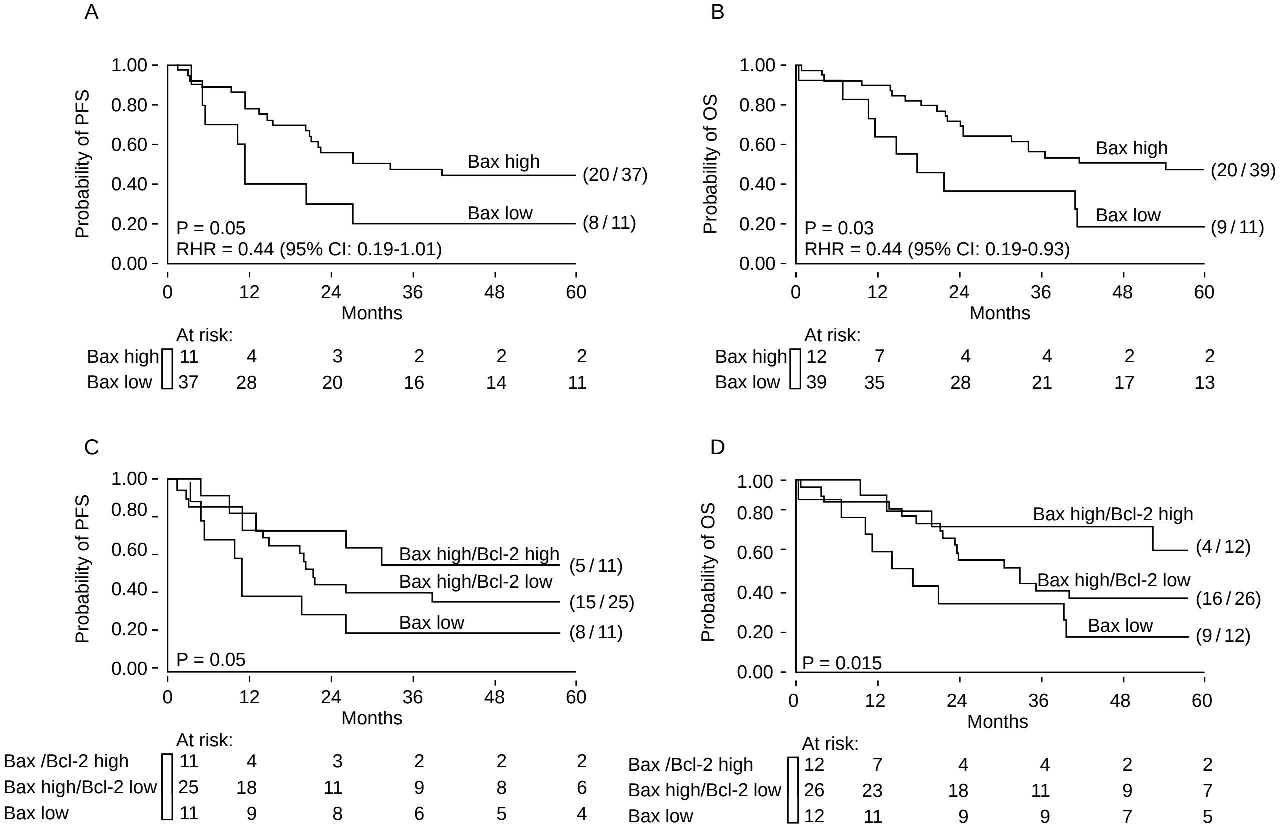

**Figure 1** Progression-free and overall survival as a function of BAX immunohistochemical status (**A** and **B**) and BAX and BCL-2 immunohistochemical status combined (**C** and **D**). The cut-off point used for BAX expression was 75% and for BCL-2 expression 40% positive tumour cells. Number in parentheses indicate number of relapses or deaths/total in each group. Numbers do not add up because of missing data for progression-free survival analysis

We have demonstrated the clinical relevance of BAX protein expression. High expression levels of BAX were found to be associated with an improved progression-free and overall survival in univariate analysis. When corrected for classical factors, BAX expression tended to be an independent factor in multivariate analysis for relapse-free survival. The clinical relevance of BAX expression has also been demonstrated by Tai et al (1998). In a smaller group of 45 ovarian cancer patients these authors showed that high BAX levels were associated with improved disease-free survival only. BAX expression was found not to be associated with overall survival, probably reflecting the short follow-up (median: 21 months) of the patients. In contrast, another study in 215 ovarian cancer patients described that BAX expression was correlated with a poor clinical outcome (Marx et al, 1997).

Since BCL-2 is a critical factor for susceptibility to an apoptotic stimulus, the ratio of BCL-2 to BAX may be even of greater importance (Oltvai and Korsmeyer, 1994). Surprisingly, in this study we observed that the combination of BAX and BCL-2 expression was a better predictor of outcome than BAX expression alone in both PFS and overall survival. Patients with both BAX and BCL-2 positive tumours showed an even better survival compared to patients with BAX positive/BCL-2 negative tumours. A recent study, on 185 stage III ovarian cancer patients by Baekelandt et al (2000), also showed that in advanced ovarian cancer the combined apoptosis related protein BAX and BCL-2 expression data was of independent prognostic significance. One explanation for this unexpected observation could be that over-expression of BCL-2 was shown to increase the half-life of BAX, suggesting a feedback mechanism that may help to maintain the ration of BCL-2 to BAX protein in physiologically appropriate ranges (Miyashita et al, 1995).

It has been suggested that BAX may be involved in the development of cisplatin resistance (Perego et al, 1996). A cisplatin-resistant ovarian cancer cell line was found to have reduced BAX mRNA levels, which is consistent with the loss of the ability of TP53 to transactivate BAX as a consequence of *TP53* mutation. We, however, have not observed an association between BAX expression levels and response to platinum-containing chemotherapy in this small group of patients. Recently, in a larger group of patients with advanced disease, Baekelandt et al (2000) also showed no association between BAX and prediction of response to cisplatin containing chemotherapy. An association between high BAX levels and improved response to combination therapy consisting of paclitaxel and cisplatin in a small group of 26 patients was observed by Tai et al (1998). A relation between BAX and paclitaxel responsiveness has further been suggested by in vitro studies showing that BAX could preferentially sensitise ovarian cancer cells to the effects of paclitaxel and vincristine, as opposed to carboplatin or ionising radiation (Strobel et al, 1996, 1997, 1998). Therefore the relation between BAX expression and response to paclitaxel needs further investigation.

In conclusion, TP53 expression but not *TP53* mutation was found to predict overall survival in ovarian cancer patients. The combined evaluation of TP53 mutation and protein expression provides additional information, especially in those patients whose tumours are negative for both expression and mutation. Furthermore, high BAX expression was found to be associated

*© 2001 Cancer Research Campaign*

with a favourable outcome in univariate analysis. The simultaneous evaluation of BAX and BCL-2 expression provides additional prognostic information when compared to BAX alone. Future studies should therefore focus on the ratio of BCL-2 to BAX in relation to clinical outcome. To establish the prognostic significance of these apoptosis related genes in malignant disease it is adviced to analyse this within large prospective and randomised studies.

### ACKNOWLEDGEMENTS

This work was supported by the Dutch Cancer Society (grant DDHK 94–840). The authors thank Henk Portengen and Marion Meijer-van Gelder for assistance in collecting tumours and patient data. We also gratefully express our thanks to the gynaecologists, internists and pathologists of the following hospitals for providing tumour specimens and clinical data: In Rotterdam: University Hospital, St. Clara Hospital, Zuiderziekenhuis, Ikazia Hospital, St Franciscus Hospital, Ruwaard van Putten Hospital, Schieland Hospital, Holy Hospital, Beatrix Hospital, and in the South Western part: Albert Schweitzer Hospital, Drechtsteden Hospital, Franciscus Hospital, Hospital Zeeuws-Vlaanderen, Hospital Walcheren, St Lievensberg Hospital, Ignatius Hospital, St van Weel-Bethesda Hospital, St Elisabeth Hospital, and Pathan.

### REFERENCES

Anttila MA, Kosma VM, Hongxiu J, Puolakka J, Juhola M, Saarikoski S and Syrjanen K (1999) p21/WAF1 expression as related to p53, cell proliferation and prognosis in epithelial ovarian cancer. *Br J Cancer* **79**: 1870–1878

Baekelandt M, Holm R, Trope CG, Nesland JM and Kristensen GB (1999a) Lack of independent prognostic significance of p21 and p27 expression in advanced ovarian cancer: an immunohistochemical study. *Clin Cancer Res* **5**: 2848–2853

Baekelandt M, Kristensen G, Nesland J, Trope C and Holm R (1999b) Clinical significance of apoptosis-related factors p53, Mdm2, and Bcl-2 in advanced ovarian cancer. *J Clin Oncol* **17**: 2061–2068

Baekelandt M, Holm R, Nesland JM, Trope CG and Kristensen GB (2000) Expression of apoptosis-related proteins is an independent determinant of patient prognosis in advanced ovarian cancer. *J Clin Oncol* **22**: 3775–3781

Barlow R, Bartholomew D and Bremner J (1993) *Statistical interference under order restrictions*. John Wiley & Sons: London

Berns EM, Klijn JG, Smid M, van Staveren IL, Look MP, van Putten WL and Foekens JA (1996) TP53 and MYC gene alterations independently predict poor prognosis in breast cancer patients. *Genes Chromosomes Cancer* **16**: 170–179

Bonetti A, Zaninelli M, Leone R, Cetto GL, Pelosi G, Biolo S, Menghi A, Manfrin E, Bonetti F and Piubello Q (1998) bcl-2 but not p53 expression is associated with resistance to chemotherapy in advanced breast cancer. *Clin Cancer Res* **4**: 2331–2336

Buttitta F, Marchetti A, Gadducci A, Pellegrini S, Morganti M, Carnicelli V, Cosio S, Gagetti O, Genazzani AR and Bevilacqua G (1997) p53 alterations are predictive of chemoresistance and aggressiveness in ovarian carcinomas: a molecular and immunohistochemical study. *Br J Cancer* **75**: 230–235

Casey G, Lopez ME, Ramos JC, Plummer SJ, Arboleda MJ, Shaughnessy M, Karlan B and Slamon DJ (1996) DNA sequence analysis of exons 2 through 11 and immunohistochemical staining are required to detect all known p53 alterations in human malignancies. *Oncogene* **13**: 1971–1981

Diebold J, Baretton G, Felchner M, Meier W, Dopfer K, Schmidt M and Lohrs U (1996) bcl-2 expression, p53 accumulation, and apoptosis in ovarian carcinomas. *Am J Clin Pathol* **105**: 341–349

Dive C and Hickman JA (1991) Drug-target interactions: only the first step in the commitment to a programmed cell death? *Br J Cancer* **64**: 192–196

Dong Y, Walsh MD, McGuckin MA, Cummings MC, Gabrielli BG, Wright GR, Hurst T, Khoo SK and Parsons PG (1997) Reduced expression of retinoblastoma gene product (pRB) and high expression of p53 are associated with poor prognosis in ovarian cancer. *Int J Cancer* **74**: 407–415

Eastman A (1987) The formation, isolation and characterization of DNA adducts produced by anticancer platinum complexes. *Pharmacol Ther* **34**: 155–166

Eastman A (1990) Activation of programmed cell death by anticancer agents: cisplatin as a model system. *Cancer Cells* **2**: 275–280

el-Deiry WS, Tokino T, Velculescu VE, Levy DB, Parsons R, Trent JM, Lin D, Mercer WE, Kinzler KW and Vogelstein B (1993) WAF1, a potential mediator of p53 tumor suppression. *Cell* **75**: 817–825

el-Deiry WS, Harper JW, O'Connor PM, Velculescu VE, Canman CE, Jackman J, Pietenpol JA, Burrell M, Hill DE and Wang Y (1994) WAF1/CIP1 is induced in p53-mediated G1 arrest and apoptosis. *Cancer Res* **54**: 1169–1174

EORTC Breast Cancer Cooperative Group (1980) Revision of the standards for the assessment of hormone receptors in human breast cancer; report of the second E.O.R.T.C. Workshop, held on 16–17 March, 1979, in the Netherlands Cancer Institute. *Eur J Cancer* **16**: 1513–1515

Ferrandina G, Fagotti A, Salerno M, Natali P, Mottolese M, Maneschi F, Pasqua AD, Benedetti-Panici P, Mancuso S and Scambia G (1999) p53 overexpression is associated with cytoreduction and response to chemotherapy in ovarian cancer. *Br J Cancer* **81**: 733–740

Gasparini G, Barbareschi M, Doglioni C, Palma PD, Mauri FA, Boracchi P, Bevilacqua P, Caffo O, Morelli L and Verderio P (1995) Expression of bcl-2 protein predicts efficacy of adjuvant treatments in operable node-positive breast cancer. *Clin Cancer Res* **1**: 189–198

Hartmann LC, Podratz KC, Keeney GL, Kamel NA, Edmonson JH, Grill JP, Su JQ, Katzmann JA and Roche PC (1994) Prognostic significance of p53 immunostaining in epithelial ovarian cancer. *J Clin Oncol* **12**: 64–69

Henriksen R, Wilander E and Oberg K (1995) Expression and prognostic significance of Bcl-2 in ovarian tumours. *Br J Cancer* **72**: 1324–1329

Herod JJ, Eliopoulos AG, Warwick J, Niedobitek G, Young LS and Kerr DJ (1996) The prognostic significance of Bcl-2 and p53 expression in ovarian carcinoma. *Cancer Res* **56**: 2178–2184

Hockenbery D, Nunez G, Milliman C, Schreiber RD and Korsmeyer SJ (1990) Bcl-2 is an inner mitochondrial membrane protein that blocks programmed cell death. *Nature* **348**: 334–336

Kaplan E and Meier P (1958) Non-parametric estimation from incomplete observations. *J Am Stat Assoc* **53**: 457–481

Kassim SK, Ali HS, Sallam MM, Fayed ST, Seada LS, abd-Elkawy E, Seada MA and Khalifa A (1999) Increased bcl-2 expression is associated with primary resistance to chemotherapy in human epithelial ovarian cancer. *Clin Biochem* **32**: 333–338

Kohler MF, Kerns BJ, Humphrey PA, Marks JR, Bast RC Jr and Berchuck A (1993) Mutation and overexpression of p53 in early-stage epithelial ovarian cancer. *Obstet Gynecol* **81**: 643–650

Kottmeier HL (1976) Presentation of therapeutic results in carcinoma of the female pelvis: experience of the Annual Report on the Results of Treatment in Carcinoma of the Uterus, Vagina, and Ovary. *Gynecol Oncol* **4**: 13–19

Koukourakis MI, Giatromanolaki A, O'Byrne KJ, Whitehouse RM, Talbot DC, Gatter KC and Harris AL (1997) Potential role of bcl-2 as a suppressor of tumour angiogenesis in non-small-cell lung cancer. *Int J Cancer* **74**: 565–570

Mano Y, Kikuchi Y, Yamamoto K, Kita T, Hirata J, Tode T, Ishii K and Nagata I (1999) Bcl-2 as a predictor of chemosensitivity and prognosis in primary epithelial ovarian cancer. *Eur J Cancer* **35**: 1214–1219

Marks JR, Davidoff AM, Kerns BJ, Humphrey PA, Pence JC, Dodge RK, Clarke-Pearson DL, Iglehart JD, Bast RC Jr and Berchuck A (1991) Overexpression and mutation of p53 in epithelial ovarian cancer. *Cancer Res* **51**: 2979–2984

Marone M, Scambia G, Mozzetti S, Ferrandina G, Iacovella S, De Pasqua A, Benedetti-Panici P and Mancuso S (1998) bcl-2, bax, bcl-XL, and bcl-XS expression in normal and neoplastic ovarian tissues. *Clin Cancer Res* **4**: 517–524

Marx D, Binder C, Meden H, Lenthe T, Ziemek T, Hiddemann T, Kuhn W and Schauer A (1997) Differential expression of apoptosis associated genes bax and bcl-2 in ovarian cancer. *Anticancer Res* **17**: 2233–2240

Miyashita T, Kitada S, Krajewski S, Horne WA, Delia D and Reed JC (1995) Overexpression of the Bcl-2 protein increases the half-life of p21 Bax. *J Biol Chem* **270**: 26049–26052

Miyashita T and Reed JC (1993) Bcl-2 oncoprotein blocks chemotherapy-induced apoptosis in a human leukemia cell line. *Blood* **81**: 151–157

Miyashita T, Krajewski S, Krajewska M, Wang HG, Lin HK, Liebermann DA, Hoffman B and Reed JC (1994) Tumor suppressor p53 is a regulator of bcl-2 and bax gene expression in vitro and in vivo. *Oncogene* **9**: 1799–1805

Miyashita T and Reed JC (1995) Tumor suppressor p53 is a direct transcriptional activator of the human bax gene. *Cell* **80**: 293–299

Neijt JP, ten Bokkel Huinink WW, van der Burg ME, van Oosterom AT, Willemse PH, Vermorken JB, van Lindert AC, Heintz AP, Aartsen E and van Lent M (1991) Long-term survival in ovarian cancer. Mature data from The

 *© 2001 Cancer Research Campaign*

Netherlands Joint Study Group for Ovarian Cancer. *Eur J Cancer* **27**: 1367–1372

Niwa K, Itoh M, Murase T, Morishita S, Itoh N, Mori H and Tamaya T (1994) Alteration of p53 gene in ovarian carcinoma: clinicopathological correlation and prognostic significance. *Br J Cancer* **70**: 1191–1197

Oltvai ZN, Milliman CL and Korsmeyer SJ (1993) Bcl-2 heterodimerizes in vivo with a conserved homolog, Bax, that accelerates programmed cell death. *Cell* **74**: 609–619

Oltvai ZN and Korsmeyer SJ (1994) Checkpoints of dueling dimers foil death wishes. *Cell* **79**: 189–192

Ozols R, Rubin S and Dembo A (1992) Principles and practice of gynecologic oncology. In: Epithelial Ovarian Cancer, Hoskins W, Perez C and Young R (eds) pp 731–781. Lippincott: Philadelphia

Perego P, Giarola M, Righetti SC, Supino R, Caserini C, Delia D, Pierotti MA, Miyashita T, Reed JC and Zunino F (1996) Association between cisplatin resistance and mutation of p53 gene and reduced bax expression in ovarian carcinoma cell systems. *Cancer Res* **56**: 556–562

Pietenpol JA, Papadopoulos N, Markowitz S, Willson JK, Kinzler KW and Vogelstein B (1994) Paradoxical inhibition of solid tumor cell growth by bcl2. *Cancer Res* **54**: 3714–3717

Righetti SC, Della Torre G, Pilotti S, Menard S, Ottone F, Colnaghi MI, Pierotti MA, Lavarino C, Cornarotti M, Oriana S, Bohm S, Bresciani GL, Spatti G and Zunino F (1996) A comparative study of p53 gene mutations, protein accumulation, and response to cisplatin-based chemotherapy in advanced ovarian carcinoma. *Cancer Res* **56**: 689–693

Rohlke P, Milde-Langosch K, Weyland C, Pichlmeier U, Jonat W and Loning T (1997) p53 is a persistent and predictive marker in advanced ovarian carcinomas: multivariate analysis including comparison with Ki67 immunoreactivity. *J Cancer Res Clin Oncol* **123**: 496–501

Schuyer M, Henzen-Logmans SC, van der Burg ME, Fieret EJ, Klijn JG, Foekens JA and Berns EM (1998) High prevalence of codon 213 ArgStop mutations of the TP53 gene in human ovarian cancer in the southwestern part of the Netherlands. *Int J Cancer* **76**: 299–303

Shelling AN, Cooke IE and Ganesan TS (1995) The genetic analysis of ovarian cancer. *Br J Cancer* **72**: 521–527

Skilling JS, Sood A, Niemann T, Lager DJ and Buller RE (1996) An abundance of p53 null mutations in ovarian carcinoma. *Oncogene* **13**: 117–123

Strobel T, Swanson L, Korsmeyer S and Cannistra SA (1996) BAX enhances paclitaxel-induced apoptosis through a p53-independent pathway. *Proc Natl Acad Sci USA* **93**: 14094–14099

Strobel T, Swanson L, Korsmeyer S and Cannistra SA (1997) Radiation-induced apoptosis is not enhanced by expression of either p53 or BAX in SW626 ovarian cancer cells. *Oncogene* **14**: 2753–2758

Strobel T, Kraeft SK, Chen LB and Cannistra SA (1998) BAX expression is associated with enhanced intracellular accumulation of paclitaxel: a novel role for BAX during chemotherapy-induced cell death. *Cancer Res* **58**: 4776–4781

Tai YT, Lee S, Niloff E, Weisman C, Strobel T and Cannistra SA (1998) BAX protein expression and clinical outcome in epithelial ovarian cancer. *J Clin Oncol* **16**: 2583–2590

van der Burg ME, Henzen-Logmans SC, Berns EM, van Putten WL, Klijn JG and Foekens JA (1996) Expression of urokinase-type plasminogen activator (uPA) and its inhibitor PAI-1 in benign, borderline, malignant primary and metastatic ovarian tumors. *Int J Cancer* **69**: 475–479

van der Zee AG, Hollema H, Suurmeijer AJ, Krans M, Sluiter WJ, Willemse PH, Aalders JG and de Vries EG (1995) Value of P-glycoprotein, glutathione S-transferase pi, c-erbB-2, and p53 as prognostic factors in ovarian carcinomas. *J Clin Oncol* **13**: 70–78

Wen WH, Reles A, Runnebaum IB, Sullivan-Halley J, Bernstein L, Jones LA, Felix JC, Kreienberg R, el-Naggar A and Press MF (1999) p53 mutations and expression in ovarian cancers: correlation with overall survival. *Int J Gynecol Pathol* **18**: 29–41

World Health Organization (1979) *WHO handbook for reporting results of cancer treatments*. Vol. no. 49. World Health Organization offset publication: Geneva

*© 2001 Cancer Research Campaign*

## References

[PDF_00778] Anttila M. A., Kosma V. M., Hongxiu J., Puolakka J., Juhola M., Saarikoski S., Syrjänen K. (1999). p21/WAF1 expression as related to p53, cell proliferation and prognosis in epithelial ovarian cancer.. Br J Cancer.

[PDF_00788] Baekelandt M., Holm R., Nesland J. M., Tropé C. G., Kristensen G. B. (2000). Expression of apoptosis-related proteins is an independent determinant of patient prognosis in advanced ovarian cancer.. J Clin Oncol.

[PDF_00781] Baekelandt M., Holm R., Tropé C. G., Nesland J. M., Kristensen G. B. (1999). Lack of independent prognostic significance of p21 and p27 expression in advanced ovarian cancer: an immunohistochemical study.. Clin Cancer Res.

[PDF_00785] Baekelandt M., Kristensen G. B., Nesland J. M., Tropé C. G., Holm R. (1999). Clinical significance of apoptosis-related factors p53, Mdm2, and Bcl-2 in advanced ovarian cancer.. J Clin Oncol.

[PDF_00793] Berns E. M., Klijn J. G., Smid M., van Staveren I. L., Look M. P., van Putten W. L., Foekens J. A. (1996). TP53 and MYC gene alterations independently predict poor prognosis in breast cancer patients.. Genes Chromosomes Cancer.

[PDF_00796] Bonetti A., Zaninelli M., Leone R., Cetto G. L., Pelosi G., Biolo S., Menghi A., Manfrin E., Bonetti F., Piubello Q. (1998). bcl-2 but not p53 expression is associated with resistance to chemotherapy in advanced breast cancer.. Clin Cancer Res.

[PDF_00800] Buttitta F., Marchetti A., Gadducci A., Pellegrini S., Morganti M., Carnicelli V., Cosio S., Gagetti O., Genazzani A. R., Bevilacqua G. (1997). p53 alterations are predictive of chemoresistance and aggressiveness in ovarian carcinomas: a molecular and immunohistochemical study.. Br J Cancer.

[PDF_00804] Casey G., Lopez M. E., Ramos J. C., Plummer S. J., Arboleda M. J., Shaughnessy M., Karlan B., Slamon D. J. (1996). DNA sequence analysis of exons 2 through 11 and immunohistochemical staining are required to detect all known p53 alterations in human malignancies.. Oncogene.

[PDF_00808] Diebold J., Baretton G., Felchner M., Meier W., Dopfer K., Schmidt M., Löhrs U. (1996). bcl-2 expression, p53 accumulation, and apoptosis in ovarian carcinomas.. Am J Clin Pathol.

[PDF_00811] Dive C., Hickman J. A. (1991). Drug-target interactions: only the first step in the commitment to a programmed cell death?. Br J Cancer.

[PDF_00813] Dong Y., Walsh M. D., McGuckin M. A., Cummings M. C., Gabrielli B. G., Wright G. R., Hurst T., Khoo S. K., Parsons P. G. (1997). Reduced expression of retinoblastoma gene product (pRB) and high expression of p53 are associated with poor prognosis in ovarian cancer.. Int J Cancer.

[PDF_00819] Eastman A. (1990). Activation of programmed cell death by anticancer agents: cisplatin as a model system.. Cancer Cells.

[PDF_00817] Eastman A. (1987). The formation, isolation and characterization of DNA adducts produced by anticancer platinum complexes.. Pharmacol Ther.

[PDF_00831] Ferrandina G., Fagotti A., Salerno M. G., Natali P. G., Mottolese M., Maneschi F., De Pasqua A., Benedetti-Panici P., Mancuso S., Scambia G. (1999). p53 overexpression is associated with cytoreduction and response to chemotherapy in ovarian cancer.. Br J Cancer.

[PDF_00835] Gasparini G., Barbareschi M., Doglioni C., Palma P. D., Mauri F. A., Boracchi P., Bevilacqua P., Caffo O., Morelli L., Verderio P. (1995). Expression of bcl-2 protein predicts efficacy of adjuvant treatments in operable node-positive breast cancer.. Clin Cancer Res.

[PDF_00839] Hartmann L. C., Podratz K. C., Keeney G. L., Kamel N. A., Edmonson J. H., Grill J. P., Su J. Q., Katzmann J. A., Roche P. C. (1994). Prognostic significance of p53 immunostaining in epithelial ovarian cancer.. J Clin Oncol.

[PDF_00842] Henriksen R., Wilander E., Oberg K. (1995). Expression and prognostic significance of Bcl-2 in ovarian tumours.. Br J Cancer.

[PDF_00844] Herod J. J., Eliopoulos A. G., Warwick J., Niedobitek G., Young L. S., Kerr D. J. (1996). The prognostic significance of Bcl-2 and p53 expression in ovarian carcinoma.. Cancer Res.

[PDF_00847] Hockenbery D., Nuñez G., Milliman C., Schreiber R. D., Korsmeyer S. J. (1990). Bcl-2 is an inner mitochondrial membrane protein that blocks programmed cell death.. Nature.

[PDF_00852] Kassim S. K., Ali H. S., Sallam M. M., Fayed S. T., Seada L. S., abd-Elkawy E., Seada M. A., Khalifa A. (1999). Increased bcl-2 expression is associated with primary resistance to chemotherapy in human epithelial ovarian cancer.. Clin Biochem.

[PDF_00856] Kohler M. F., Kerns B. J., Humphrey P. A., Marks J. R., Bast R. C., Berchuck A. (1993). Mutation and overexpression of p53 in early-stage epithelial ovarian cancer.. Obstet Gynecol.

[PDF_00859] Kottmeier H. L. (1976). Presentation of therapeutic results in carcinoma of the female pelvis: experience of the Annual Report on the Results of Treatment in Carcinoma of the Uterus, Vagina, and Ovary.. Gynecol Oncol.

[PDF_00862] Koukourakis M. I., Giatromanolaki A., O'Byrne K. J., Whitehouse R. M., Talbot D. C., Gatter K. C., Harris A. L. (1997). Potential role of bcl-2 as a suppressor of tumour angiogenesis in non-small-cell lung cancer.. Int J Cancer.

[PDF_00866] Mano Y., Kikuchi Y., Yamamoto K., Kita T., Hirata J., Tode T., Ishii K., Nagata I. (1999). Bcl-2 as a predictor of chemosensitivity and prognosis in primary epithelial ovarian cancer.. Eur J Cancer.

[PDF_00869] Marks J. R., Davidoff A. M., Kerns B. J., Humphrey P. A., Pence J. C., Dodge R. K., Clarke-Pearson D. L., Iglehart J. D., Bast R. C., Berchuck A. (1991). Overexpression and mutation of p53 in epithelial ovarian cancer.. Cancer Res.

[PDF_00873] Marone M., Scambia G., Mozzetti S., Ferrandina G., Iacovella S., De Pasqua A., Benedetti-Panici P., Mancuso S. (1998). bcl-2, bax, bcl-XL, and bcl-XS expression in normal and neoplastic ovarian tissues.. Clin Cancer Res.

[PDF_00877] Marx D., Binder C., Meden H., Lenthe T., Ziemek T., Hiddemann T., Kuhn W., Schauer A. (1997). Differential expression of apoptosis associated genes bax and bcl-2 in ovarian cancer.. Anticancer Res.

[PDF_00880] Miyashita T., Kitada S., Krajewski S., Horne W. A., Delia D., Reed J. C. (1995). Overexpression of the Bcl-2 protein increases the half-life of p21Bax.. J Biol Chem.

[PDF_00885] Miyashita T., Krajewski S., Krajewska M., Wang H. G., Lin H. K., Liebermann D. A., Hoffman B., Reed J. C. (1994). Tumor suppressor p53 is a regulator of bcl-2 and bax gene expression in vitro and in vivo.. Oncogene.

[PDF_00883] Miyashita T., Reed J. C. (1993). Bcl-2 oncoprotein blocks chemotherapy-induced apoptosis in a human leukemia cell line.. Blood.

[PDF_00888] Miyashita T., Reed J. C. (1995). Tumor suppressor p53 is a direct transcriptional activator of the human bax gene.. Cell.

[PDF_00897] Niwa K., Itoh M., Murase T., Morishita S., Itoh N., Mori H., Tamaya T. (1994). Alteration of p53 gene in ovarian carcinoma: clinicopathological correlation and prognostic significance.. Br J Cancer.

[PDF_00903] Oltvai Z. N., Korsmeyer S. J. (1994). Checkpoints of dueling dimers foil death wishes.. Cell.

[PDF_00900] Oltvai Z. N., Milliman C. L., Korsmeyer S. J. (1993). Bcl-2 heterodimerizes in vivo with a conserved homolog, Bax, that accelerates programmed cell death.. Cell.

[PDF_00908] Perego P., Giarola M., Righetti S. C., Supino R., Caserini C., Delia D., Pierotti M. A., Miyashita T., Reed J. C., Zunino F. (1996). Association between cisplatin resistance and mutation of p53 gene and reduced bax expression in ovarian carcinoma cell systems.. Cancer Res.

[PDF_00912] Pietenpol J. A., Papadopoulos N., Markowitz S., Willson J. K., Kinzler K. W., Vogelstein B. (1994). Paradoxical inhibition of solid tumor cell growth by bcl2.. Cancer Res.

[PDF_00915] Righetti S. C., Della Torre G., Pilotti S., Ménard S., Ottone F., Colnaghi M. I., Pierotti M. A., Lavarino C., Cornarotti M., Oriana S. (1996). A comparative study of p53 gene mutations, protein accumulation, and response to cisplatin-based chemotherapy in advanced ovarian carcinoma.. Cancer Res.

[PDF_00920] Röhlke P., Milde-Langosch K., Weyland C., Pichlmeier U., Jonat W., Löning T. (1997). p53 is a persistent and predictive marker in advanced ovarian carcinomas: multivariate analysis including comparison with Ki67 immunoreactivity.. J Cancer Res Clin Oncol.

[PDF_00924] Schuyer M., Henzen-Logmans S. C., van der Burg M. E., Fieret E. J., Klijn J. G., Foekens J. A., Berns E. M. (1998). High prevalence of codon 213Arg-->Stop mutations of the TP53 gene in human ovarian cancer in the southwestern part of The Netherlands.. Int J Cancer.

[PDF_00928] Shelling A. N., Cooke I. E., Ganesan T. S. (1995). The genetic analysis of ovarian cancer.. Br J Cancer.

[PDF_00930] Skilling J. S., Sood A., Niemann T., Lager D. J., Buller R. E. (1996). An abundance of p53 null mutations in ovarian carcinoma.. Oncogene.

[PDF_00938] Strobel T., Kraeft S. K., Chen L. B., Cannistra S. A. (1998). BAX expression is associated with enhanced intracellular accumulation of paclitaxel: a novel role for BAX during chemotherapy-induced cell death.. Cancer Res.

[PDF_00932] Strobel T., Swanson L., Korsmeyer S., Cannistra S. A. (1996). BAX enhances paclitaxel-induced apoptosis through a p53-independent pathway.. Proc Natl Acad Sci U S A.

[PDF_00935] Strobel T., Swanson L., Korsmeyer S., Cannistra S. A. (1997). Radiation-induced apoptosis is not enhanced by expression of either p53 or BAX in SW626 ovarian cancer cells.. Oncogene.

[PDF_00942] Tai Y. T., Lee S., Niloff E., Weisman C., Strobel T., Cannistra S. A. (1998). BAX protein expression and clinical outcome in epithelial ovarian cancer.. J Clin Oncol.

[PDF_00953] Wen W. H., Reles A., Runnebaum I. B., Sullivan-Halley J., Bernstein L., Jones L. A., Felix J. C., Kreienberg R., el-Naggar A., Press M. F. (1999). p53 mutations and expression in ovarian cancers: correlation with overall survival.. Int J Gynecol Pathol.

[PDF_00825] el-Deiry W. S., Harper J. W., O'Connor P. M., Velculescu V. E., Canman C. E., Jackman J., Pietenpol J. A., Burrell M., Hill D. E., Wang Y. (1994). WAF1/CIP1 is induced in p53-mediated G1 arrest and apoptosis.. Cancer Res.

[PDF_00822] el-Deiry W. S., Tokino T., Velculescu V. E., Levy D. B., Parsons R., Trent J. M., Lin D., Mercer W. E., Kinzler K. W., Vogelstein B. (1993). WAF1, a potential mediator of p53 tumor suppression.. Cell.

[PDF_00945] van der Burg M. E., Henzen-Logmans S. C., Berns E. M., van Putten W. L., Klijn J. G., Foekens J. A. (1996). Expression of urokinase-type plasminogen activator (uPA) and its inhibitor PAI-1 in benign, borderline, malignant primary and metastatic ovarian tumors.. Int J Cancer.

[PDF_00949] van der Zee A. G., Hollema H., Suurmeijer A. J., Krans M., Sluiter W. J., Willemse P. H., Aalders J. G., de Vries E. G. (1995). Value of P-glycoprotein, glutathione S-transferase pi, c-erbB-2, and p53 as prognostic factors in ovarian carcinomas.. J Clin Oncol.

